# How Well Do All Patient Refined–Diagnosis-Related Groups Explain Costs of Pediatric Cancer Chemotherapy Admissions in the United States?

**DOI:** 10.1200/JOP.2015.010330

**Published:** 2016-04-26

**Authors:** Heidi Russell, Andrew Street, Vivian Ho

**Affiliations:** Baylor College of Medicine; Rice University, Houston, TX; and University of York, York, United Kingdom

## Abstract

**Purpose:**

State-based Medicaid programs have begun using All Patient Refined–Diagnosis-Related Groups (APR-DRGs) to determine hospital reimbursement rates. Medicaid provides coverage for 45% of childhood cancer admissions. This study aimed to examine how well APR-DRGs reflect admission costs for childhood cancer chemotherapy to inform clinicians, hospitals, and policymakers in the wake of policy changes.

**Methods:**

We identified 25,613 chemotherapy admissions in the 2009 Kids’ Inpatient Database. To determine how well APR-DRGs explain costs, we applied a hierarchic linear regression model of hospital costs, allowing for a variety of patient, hospital, and geographic confounders.

**Results:**

APR-DRGs proved to be the most important predictors of admission costs (*P* < .001), with costs increasing by DRG severity code. Diagnosis, age, and hospital characteristics also predicted costs above and beyond those explained by APR-DRGs. Compared with admissions for patients with acute lymphoblastic leukemia, costs of admissions for patients with acute myelomonocytic leukemia were 82% higher; non-Hodgkin lymphoma, 20% higher; Hodgkin lymphoma, 25% lower; and CNS tumors, 27% lower. Admissions for children who were 10 years of age or older cost 26% to 35% more than admissions for infants. Admissions to children’s hospitals cost 46% more than admissions to other hospital types.

**Conclusion:**

APR-DRGs developed for adults are applicable to childhood cancer chemotherapy but should be refined to account for cancer diagnosis and patient age. Possible policy and clinical management changes merit further study to address factors not captured by APR-DRGs.

## INTRODUCTION

Diagnosis-related groups (DRGs) are designed to classify clinically similar groups of patients with similar resource requirements.^1^ Since the early 1980s, the Centers for Medicare and Medicaid Services has used DRGs as part of a prospective reimbursement system under Medicare. State-based Medicaid systems historically used combinations of cost-based and managed care capitation to pay for hospital care. In 2013, states began shifting Medicaid inpatient reimbursement to prospective strategies using All Patient Refined–DRGs (APR-DRGs).^2-7^ APR-DRGs further classify admissions within a DRG into one of four severity codes after considering factors such as severity of the primary illness, comorbidities and secondary diagnoses, and services used during the admission. A reimbursement rate is assigned to each DRG plus severity code. Of course, some patients will actually have higher costs than expected; costs will be lower for others. The differences cancel one another out as long as the mix of patients within each DRG is random, and DRGs accurately reflect clinical resource utilization.

Most DRGs are the same for children and adults, with the exception of acute leukemia (DRG 690) and groups for neonatal conditions. To date, little examination of the adequacy of APR-DRGs to predict resource utilization in childhood admissions has occurred. Childhood cancer admissions cost approximately $1.9 billion nationally in 2009, nearly 11% of all inpatient costs for non-newborn children younger than age 21 years.^8,9^ Almost half of childhood cancer admissions are for chemotherapy.^8^ Because Medicaid provides medical coverage for 45% of pediatric cancer admissions,^10^ the frequency and high resource utilization of chemotherapy admissions makes this an area of potential fiscal risk if APR-DRGs and actual utilization are not aligned.

Our overall goal is to provide guidance for clinicians, hospitals, and policymakers in view of policy to reimburse Medicaid admissions using APR-DRGs. In this report, we analyze the ability of APR-DRGs to predict inpatient costs among children with cancer admitted for chemotherapy by examining national samples of hospital admissions for 2009. The analysis controlled for diagnosis-, patient-, and hospital-level factors, and we estimated the effect each set of factors had on admission costs.

## METHODS

### Study Sample

A cancer cohort was identified from the 2009 Kids’ Inpatient Database (KID), compiled by the Healthcare Cost and Utilization Project, and sponsored by the US Agency for Healthcare Research and Quality.^11^ The Institutional Review Board of Baylor College of Medicine considered this research exempt from review.

KID samples approximately 80% of pediatric discharges from community hospitals; the 2009 sampling included 3.4 million deidentified admission records for patients younger than the age of 21 years from 5,128 hospitals in 44 states. For each record, the database contains APR-DRGs as assigned by the 3M grouper version (3M Health Information Systems, Salt Lake City, UT) in use at discharge^12^ and up to 25 discharge diagnoses and 15 procedures coded according to the International Classification of Diseases, Ninth Revision, Clinical Modification (ICD-9-CM). KID also includes patient age, sex, health care payer, and a measure of the patient’s income status by zip code. States optionally provide race/ethnicity, income status, and hospital characteristics; any systematically omitted data were coded as missing.

Cancer-related admissions were identified from KID by an ICD-9-CM code for cancer in any discharge diagnosis.^9^ Admissions to deliver chemotherapy were identified as admissions with a chemotherapy or immunotherapy procedure on or before hospital day 2 or with chemotherapy as the primary diagnostic code.^9^ Each admission was assigned to one of seven cancer diagnoses by ICD-9-CM codes or categorized as dual if it had more than one cancer diagnosis: acute lymphoblastic leukemia (ALL), acute myelomonocytic leukemia (AML), non-Hodgkin lymphomas, Hodgkin lymphomas, CNS tumors, bone and joint tumors, and soft tissue tumors. Discharge codes used to assign diagnoses are listed in Appendix Table A1 (online only). We excluded chronic forms of leukemia, benign or premalignant neoplasms, and admissions associated with a hematopoietic stem-cell transplantation procedure.

### Hospital Costs and Characteristics

Total admission charges in KID were converted to costs using cost-to-charge ratios.^13^ KID contains hospital characteristics including a hospital’s designation by the Children’s Hospitals of America, teaching status, location (urban or rural), and state. For this analysis, admissions at children’s specialty hospitals were excluded because they accounted for less than 1% of all admissions. KID categorizes hospitals as small, medium, or large by considering number of beds, urban or rural location, and state. This hospital-size category caused unacceptable multicollinearity with other hospital descriptors. Instead, the number of chemotherapy admissions for each hospital was used as a descriptor of cancer volume, with categorization into five groups using cut point selection.^14^ States were grouped into nine divisions defined by the US Census Bureau.^15^

### APR-DRGs

The most frequent APR-DRGs were identified from the chemotherapy admissions. APR-DRGs, including severity codes that individually represented more than 1% of admissions, were included as individual explanatory variables. APR-DRGs below this threshold were combined into a single “other” category.

### Statistical Analysis

The analytic purpose was to determine how well APR-DRGs explain the total cost of an individual admission relative to other patient or hospital characteristics. We estimated linear hierarchic regression models, recognizing that admissions (level one) are clustered within hospitals (level two). Four linear models with random hospital effects were estimated: only APR-DRG variables, only patient characteristics, only hospital characteristics, and all characteristics of interest.^16^ Postestimation Wald tests were used to compare factors within categorical variables.

We tested the robustness of our models under alternative scenarios. Admissions with systematically omitted race/ethnicity or hospital characteristics were removed from all four models. The effect of state-level variation was evaluated by first including an indicator for individual states and then grouping states into four regions.^15^ The sensitivity of the model to extreme costs was assessed by excluding admissions costing more than $85,000 (three standard deviations above the mean cost) or less than $1,200. Another analysis examined the sensitivity of the model when limited to only DRG 693. Finally, the robustness of the model was tested after identifying admissions associated with an infection,^9^ a possible quality indicator.

We performed exploratory analyses on two descriptors that significantly affected costs after controlling for APR-DRGs in the full model: diagnosis and age. To explore the variation in the distribution of diagnoses and admission costs among hospitals, we first identified all hospitals with more than 25 chemotherapy admissions, with 65% to 75% of these admissions classified as APR-DRG 693-2 (n = 60). For each hospital, we calculated the ratio of combined ALL, bone or joint tumor, or soft tissue tumor admissions to AML admissions within this APR-DRG. We then calculated the mean and range of costs of AML admissions within APR-DRG 693-2 from the four hospitals (of the 60) with more than 10 AML admissions. To explore the effects of age on costs, we limited the full model to each of the three most common diagnoses: ALL, bone and joint tumors, and soft tissue tumors. We tested each limited model with and without interactions between age and APR-DRG.

## RESULTS

### Descriptive Results

The characteristics of 23,846 admissions for chemotherapy are listed in Table 1. APR-DRG 693 (chemotherapy) accounted for 90% of all admissions, and most of these were considered level two in severity. The only other APR-DRG accounting for more than 1% of admissions was 690-2 (acute leukemia), severity level two. The “other” group included 138 DRGs (not considering severity level), ranging in frequency from one to 366 admissions.

**Table 1.** Characteristics of Chemotherapy Admissions in 2009 Kids Inpatient Database

Characteristic	Admissions		Cost (2009 US$)	
	No.	%	Mean	SD
Admissions				
Total	23,846	100	12,895	23,523
APR-DRG severity level				
1: 693- 1	1,335	5.6	6,523	4,906
2: 693- 2	16,910	70.9	8,733	8,886
3: 693- 3	2,837	11.9	18,868	23,963
4: 693- 4	352	1.5	66,435	62,623
5: 690- 2	301	1.3	32,957	29,114
6: all others	2,111	8.9	30,456	54,808
Diagnosis				
ALL	5,394	22.6	15,107	28,635
AML	1,459	6.1	42,408	53,197
NHL	1,568	6.6	12,984	18,773
Hodgkin lymphoma	1,082	4.5	7,858	9,774
CNS	2,245	9.4	9,244	20,998
Bone or joint tumor	5,800	24.3	10,008	9,916
Soft tissue tumor	6,173	25.9	8,706	10,970
Dual	125	0.5	21,916	36,706
Sex				
Male	13,804	57.9	12,714	24,234
Age, years				
< 1	758	3.2	15,727	35,989
1-4	5,052	21.1	12,193	22,229
5-9	4,940	20.7	11,437	21,332
10-14	5,837	24.5	13,253	22,687
≥ 15	7,259	30.4	13,793	24,737
Race				
White	11,393	47.8	11,785	21,849
Black	2,440	10.2	11,630	21,659
Hispanic	5,207	21.8	14,974	25,272
Other	1,949	8.2	15,031	29,403
Missing	2,857	12.0	13,157	23,322
Payer				
Public	9,188	38.5	13,241	24,805
Private	12,721	53.3	12,610	22,262
Other	1,937	8.1	13,126	25,267
Zip code income quartile				
1 (lowest)	5,880	24.7	12,140	23,597
2	6,075	25.5	12,775	23,886
3	5,707	24.0	12,524	21,109
4 (highest)	5,656	23.7	14,077	25,107
Missing	528	2.2	14,034	25,216
Hospitals				
Type of hospital				
Not identified as children’s hospital	3,100	13.0	11,373	18,261
Free-standing children’s hospital	6,769	28.4	17,539	27,575
Children’s unit in general hospital	9,888	41.5	10,363	20,524
Missing	4,089	17.2	12,487	25,378
No. of chemotherapy discharges				
≤ 25	1,041	4.4	9,971	13,124
> 25 to ≤ 100	4,330	18.2	9,246	17,073
> 100 to ≤ 200	7,798	32.7	11,711	21,507
> 200 to ≤ 300	4,041	17.0	14,601	25,490
> 300	6,636	27.8	16,089	28,436
Location/teaching status				
Rural	126	0.5	6,561	7,691
Urban nonteaching	1,807	7.6	9,632	14,952
Urban teaching	17,995	75.5	13,337	23,747
Missing	3,918	16.4	12,574	25,801
Geographic division				
Northeast	921	3.9	14,423	32,386
Middle Atlantic	2,300	9.7	12,320	25,117
East North Central	3,457	14.5	11,190	18,763
West North Central	1,796	7.5	13,096	23,966
South Atlantic	4,152	17.4	10,020	17,502
East South Central	1,749	7.3	7,649	8,928
West South Central	3,437	14.4	14,326	28,268
Mountain	1,168	4.9	13,198	18,998
Pacific	4,866	20.4	17,273	27,793

Abbreviations: ALL, acute lymphoblastic leukemia; AML, acute myelomonocytic leukemia; APR-DRG, All Patient Refined–Diagnosis-Related Group; NHL, Non-Hodgkin lymphoma; SD, standard deviation.

The sample included admissions from 369 hospitals. Most hospitals were identified as not a children’s hospital (n = 207; 56%) or a children’s unit in a general hospital (n = 95; 26%). The majority of these hospitals (n = 254; 68%) were considered large hospitals by the KID definition (ie, urban teaching hospitals with > 325 beds),^11^ but each accounted for fewer than 100 childhood chemotherapy admissions. Sixteen (59%) of the 27 freestanding children’s hospitals accounted for more than 200 chemotherapy admissions each; however, only five (19%) were considered large. Characteristics of the remaining 44 hospitals were omitted; 36 (82%) of these included fewer than 100 chemotherapy admissions each.

### Regression Results

Table 2 lists the results of each of the four models. The model that included only the effects of APR-DRGs explained 16% (*r*^2^ = 0.162) of the variation in costs—more than the variation explained by patient characteristics only (13%) or by hospital characteristics only (9%). The full model that combined all of the variables had much higher explanatory power (33%). Most of the coefficients that proved significant (*P* < .001) in the three partial models remained so in the full model, although they were somewhat smaller, as would be expected. This implies that these significant variables were individually capturing important information explaining variation in costs among patients.

**Table 2.** Results of Hierarchic Models

Variable	Log of Total Admission Cost			
	M_APR-DRG_	M_Patient_	M_Hospital_	M_Full_
Admissions				
APR-DRG				
693.1	Ref			Ref
693.2	0.231*			0.147*
693.3	0.774*			0.608*
693.4	2.092*			1.630*
690.2	1.474*			1.348*
Other	0.977*			0.888*
Cancer diagnosis				
ALL		Ref		Ref
AML		0.966*		0.810*
NHL		0.058		0.200*
Hodgkin lymphoma		−0.448*		−0.249*
CNS		−0.410*		−0.268*
Bone or joint tumor		−0.155†		0.048
Soft tissue tumor		−0.252*		−0.047
Dual tumors		0.237†		0.213†
Sex				
Male		−0.030‡		−0.025
Age, years				
< 1		Ref		Ref
1 to < 5		−0.010		0.018
5 to < 10		0.019‡		0.078
10 to < 15		0.204*		0.257*
≥ 15		0.288*		0.340*
Race				
White		Ref		Ref
Black		0.007		−0.001
Hispanic		0.028		0.026
Other		−0.024		−0.012
Missing		0.022		−0.002
Payer				
Public		Ref		Ref
Private		−0.004		−0.010
Other		0.013		0.013
Zip code income level				
Lowest		Ref		Ref
Low		−0.039‡		−0.032
High		−0.036		−0.031
Highest		−0.030		−0.025
Missing		−0.019		−0.014
NACHRI hospitals				
Type of hospital				
Not identified as children’s hospital			Ref	Ref
Freestanding children’s hospital			0.423*	0.465*
Children’s unit in general hospital			−0.067	−0.047
Missing			0.441	0.308
No. of chemotherapy discharges				
≤ 25			Ref	Ref
> 25 to ≤ 100			−0.070	0.061
>100 to ≤ 200			0.054	0.182‡
>200 to ≤ 300			0.116	0.233‡
> 300			0.120	0.229‡
Location/teaching status				
Rural			Ref	Ref
Urban nonteaching			0.259‡	0.166
Urban teaching			0.235‡	0.145
Missing			−0.178	−0.100
Geographic division				
Northeast			Ref	Ref
Middle Atlantic			−0.217	−0.150
East North Central			−0.217	−0.145
West North Central			−0.169	−0.169
South Atlantic			−0.180	−0.131
East South Central			−0.293	−0.223
West South Central			−0.101	−0.037
Mountain			−0.126	−0.152
Pacific			0.063	0.100
Intercept	8.427*	8.746*	8.677*	8.101*
No. of admissions	23,846	23,846	23,846	23,846
No. of hospitals	369	369	369	369
* r*^2^	0.162	0.126	0.088	0.334

Abbreviations: ALL, acute lymphoblastic leukemia; AML, acute myelomonocytic leukemia; APR-DRG, All Patient Refined–Diagnosis-Related Group; M_APR-DRG_, model of APR-DRG variables only; M_Patient_, model of diagnosis and patient characteristics only; M_Hospital_, model of hospital characteristics only; M_Full_, full model containing all admission characteristics; NACHRI, National Association of Children’s Hospitals and Related Institutions; NHL, non-Hodgkin lymphoma; Ref, referent.

* *P* < .001.

† *P* < .01.

‡ *P* < .05.

Focusing on the full model estimates, the allocated APR-DRGs were the explanatory variables with the greatest coefficients. Costs for those allocated to DRG 693 increased by severity code. Compared with those allocated to 693.1, admissions allocated to 693.2 were 15% more costly; those allocated to 693.3 were 61% more costly, and those allocated to 693.4 were 163% more costly. Admissions allocated to APR-DRG 690.2 were 135% more costly than 693.1 admissions.

Costs varied for admissions according to cancer diagnosis, over and above the APR-DRG allocation of the admission. Average costs were similar for admissions for ALL, bone or joint tumors, or soft tissue tumors, each comprising one-quarter of all admissions. Compared with admission costs for ALL, costs were higher for AML (81%) and non-Hodgkin lymphoma (20%) admissions but lower for Hodgkin lymphoma (−25%) and CNS (−27%) admissions. The size and significance of the estimates for the effects of each diagnosis changed between the model including patient characteristics only and the full model, implying that the APR-DRGs were capturing some, but not all, of the effects of diagnosis on admission costs.

Age was another important predictor of costs. Admissions for children 10 to 14 years of age had 25% higher costs than admissions for infants; those for children older than 15 years had 35% higher costs. No other patient characteristic significantly explained costs.

Of the hospital characteristics, the only significant factor was whether the admission was to a freestanding children’s hospital. Such admissions had costs that were, on average, 46% higher than those for other hospital types. The number of chemotherapy admissions had a modest but significant effect on admission costs in the full model but not in the model including hospital characteristics only. Stepwise removal of patient descriptors demonstrated this variable was affected by collinearity with both age and diagnosis.

### Sensitivity Analyses

Effects of APR-DRGs on costs were unchanged after removing admissions with systematically missing race/ethnicity, economic status, or hospital information and when geography was grouped by state or region. The effects of the severity codes within DRG 693 were also unchanged when the sample was restricted to admissions allocated to this DRG. Approximately 3% of all chemotherapy admissions had extremely high or low costs. Removal of these admissions decreased the effects of APR-DRG 693.4 to 1.378, APR-DRG other to 0.710, and AML to 0.689, although all three remained significant. These three admission descriptors were over-represented (25%, 44%, and 48%, respectively) in the extremely high–cost admissions. We identified 5,824 admissions with one or more discharge diagnosis codes for infection. Infection status was associated with 32% higher costs, with its inclusion reducing the size of the coefficients associated with the APR-DRG by 10% to 20%.

### Diagnosis and Age

**FIG 1.**
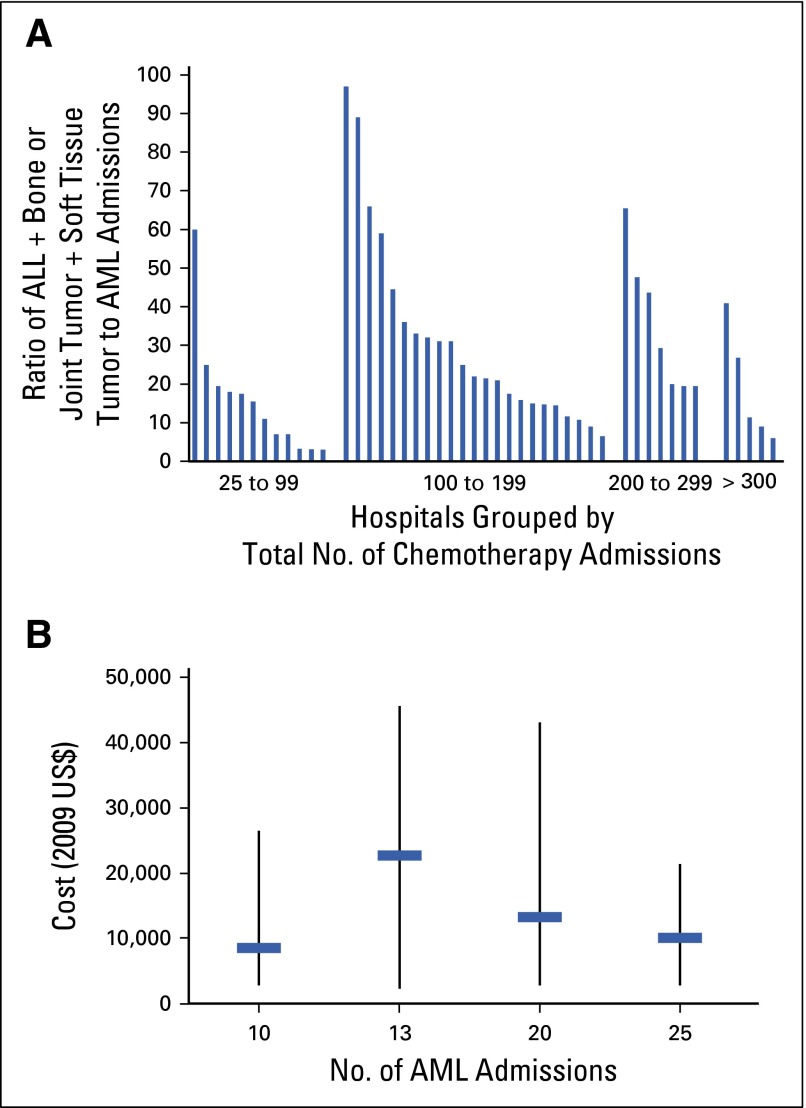
Variation in (A) ratio of combined acute lymphoblastic leukemia (ALL), bone or joint tumor, or soft tissue tumor to acute myelomonocytic leukemia (AML) chemotherapy admissions by hospital and (B) mean (horizontal bar) and range (vertical bar) of costs per AML admission in hospitals with 10 or more AML admissions; admissions limited to All Patient Refined–Diagnosis-Related Group 693.2.

Because AML admissions were infrequent but had a large effect on admission costs, the burden of the higher costs may be unevenly distributed among hospitals. Of 60 hospitals with 65% to 75% of admissions classified as APR-DRG 693.2, 13 (22%) had no AML admissions. For the remaining hospitals, ratios ranged from 97:1 to 3:1 ALL, bone or joint tumor, or soft tissue tumor admissions for every AML admission, suggesting some hospitals admit a larger proportion of patients with AML than others. Figure 1A demonstrates that this variation existed irrespective of hospital chemotherapy volume. Figure 1B depicts the large variation in costs of AML admissions among four hospitals, suggesting this grouping may not fully represent variations of resource utilization for this diagnosis.

Older age was associated with higher-cost admissions. Age also factors into risk stratification and treatment decisions in many diagnoses, so we ran the full model limited to each diagnosis group. When the model included only ALL admissions, admissions for infants and children age older than 15 years were significantly more costly than those of other age groups. Costs increased steadily by age for soft tissue tumor admissions but not significantly for bone or joint tumor admissions. Infants, our reference group, represented less than 1% of bone or joint tumor admissions. When we applied postestimation Wald testing, each bone or joint tumor age category differed significantly from one another, with the exception of those age 1 to younger than 5 compared with age 5 to younger than 10 years. There were no significant interactions between APR-DRG and age categories.

## DISCUSSION

We analyzed the ability of APR-DRGs to predict the costs of chemotherapy admissions for childhood cancers from a nationally representative data set. In this model, most admissions were classified as DRG 693. Increasing APR-DRG severity codes within this DRG were associated with increasing costs, suggesting that severity codes capture increasing intensity of chemotherapy admissions. We also identified factors potentially predictive of the admission costs currently not adequately captured by DRGs, notably cancer diagnosis and age. Medicaid systems have adopted prospective reimbursement schemes, in part, to drive efficiency. Historically, such adoptions have also been associated with hospitals or providers decreasing services because of underpayment.^17-19^ In light of this policy change, it will be crucial to identify which hospitals are experiencing the most significant drops in reimbursement and whether these drops are associated with reductions in treatment or worst outcomes.

A small but growing body of literature is identifying limitations of DRG algorithms in capturing important clinical factors. Parnell et al^20^ found that congenital cardiac disease was systematically misclassified by APR-DRGs in the United States, with potential effects on hospital-level mortality metrics. In a single-institution study, DRGs underidentified trauma patients, with resulting financial misappropriation.^21^ The EuroDRG research group classified 10 encounter types by 11 DRG algorithms and found significant variation across countries.^22-25^ Continued analysis of how well DRGs predict actual patient care is required to ensure that DRGs adequately reflect patient characteristics and treatments in both pediatric and adult admissions, particularly in light of recent incorporation of ICD-10-CM codes. Although the Centers for Medicare and Medicaid Services predicts minimal impact on overall payments as DRG algorithms change from ICD-9-CM to ICD-10-CM codes, there may be a varying impact at the hospital level.^26^

Our model suggests that costs associated with cancer diagnosis are not completely captured by the APR-DRGs. Diagnosis determines the chemotherapy regimen and, in turn, toxicity and supportive measures.^27-30^ Childhood AML treatment has been associated with high toxicities^31,32^ and prolonged admissions.^33^ APR-DRG reimbursement schemes will likely encourage clinicians and researchers to reconsider some components of AML treatment, but significant changes require time to ensure that patient outcomes and safety are maintained. If cancer diagnoses within APR-DRGs are not adequately accounted for in reimbursement policies, hospitals with more AML admissions may be at higher fiscal risk.

We performed age and diagnosis subanalyses that demonstrated how difficult it is to make specific policy or practice recommendations with respect to age. Age affected costs differently when we limited our samples to a single diagnosis, suggesting that diagnosis- and risk-specific treatments may play a role. For example, infants and children older than 10 years of age with ALL are considered at higher risk for recurrence and treated with more-intense chemotherapy^34^ than children of other ages. This clinical risk stratification may underlie the higher costs for infants and children with ALL who are older than 15 years. Alternatively, chemotherapy is dosed according to weight, which naturally increases with age during childhood. For higher-cost pharmaceuticals, the difference between infants and teenagers for the same agent may be substantial.^27^ The drivers of this cost difference could be studied further in data sets that allow identification of possible cost drivers.

We found that freestanding children’s hospitals had significantly higher costs on average than other types of hospitals. Previous literature suggests this difference may not be limited to chemotherapy, because similar effects have been identified in cardiac transplantation,^35^ sickle cell disease,^36^ and sinusitis.^37^ Direct comparison of our results with these studies is difficult because they used hospital charges rather than costs in their analyses. Romley et al^38^ linked higher costs to better outcomes for congenital heart disease surgery in this same hospital cohort. We cannot make such a direct link, because cancer outcomes are measured at a time quite distant from chemotherapy delivery and are influenced by many factors. In this data set, freestanding children’s hospitals each cared for a large volume of patients but were overall small compared with other hospital types. The average freestanding children’s hospital has approximately 280 beds (range, 78 to 564 beds), with specialized care focused on a limited population and an emphasis on medically complex cases.^39^ This has the potential to put freestanding children’s hospitals at a disadvantage in both economies of scale and economies of scope. Hospitals treating adults, already subject to DRG reimbursement, might have found cost-saving measures in cross-hospital services that lowered the overall cost for pediatric admissions. Only 27 of the 43 freestanding children’s hospitals in the United States were identified in this model, and this subset may differ from those excluded because their states do not participate in KID. The hospital characteristics included in KID are general descriptors; future studies including more detailed hospital information will be important for hospital administrators and policymakers desiring to maintain the critical role these institutions play in medical care for children.

Several limitations arise from the use of the KID data set. First, a lack of pharmaceutical data prevented investigation of the chemotherapy delivered. Second, the data set lacks patient identification. A child may be admitted multiple times during the course of his or her cancer treatment, but repeat admissions could not be tracked. Therefore, the influences of heavier users of medical care could not be accounted for outside of the sociodemographic characteristics we included. However, because reimbursement occurs by admission rather than the complete course of treatment, this methodologic limitation actually reflects payment arrangements. We were unable to determine actual reimbursement arrangements for each hospital. Each state determines its own reimbursement weights for APR-DRG and severity-level admissions, adjustments for local wages or participation in medical education, and provisions for admissions with extremely high costs or prolonged hospitalizations.^3-6^ These limitations can be addressed in other smaller data sets and were offset by the broad geographic and hospital sampling of KID. Finally, KID only provides a total charge for the admission, a markup of costs determined by each hospital.^40^ We used cost-to-charge ratios that represented markup practices aggregated across the entire hospital; this may not accurately reflect the costs of individual departments. However, past studies have suggested that the difference is generally less than 10%^41^ and that similar admissions, such as those within a DRG group, are likely to use similar resources across organizations, further decreasing between-hospital variation.^41,42^

In conclusion, APR-DRGs reflected much of the differences in costs of childhood cancer chemotherapy admissions. However, this analysis suggests that other factors are also important in predicting costs, most notably diagnosis and patient age. These should be considered in future design of APR-DRGs to make them more resource homogeneous. As health care resources become increasingly constrained, understanding where and how care and reimbursement currently misalign can identify target areas for future study and refinement.

## Supplementary Material

Publisher's Note

## Appendix

**Table A1.** Discharge Diagnosis Codes Used to Categorize Admissions

Diagnosis Category	Discharge Diagnosis Code
ALL	ICD.9-CM 204.X*
AML	ICD.9-CM 205.X, 206.X, 207.X*
NHL	ICD.9-CM 200.X, 202.X
Hodgkin lymphoma	ICD.9-CM 201.X
CNS and brain tumors	ICD.9-CM 191.X, 192.X, 194.3, 194.4
Bone and joint tumors	ICD.9-CM 170.X
Soft tissue tumors	ICD.9-CM 140.X, 150.X, 171.X to 175.X, 179.X to 189.X, 190.X, 193.X, 194.0 to 194.29, 194.5 to 194.9, 195.X, 209.0 to 209.3
Infection	CCS† 1 to 4, 7, 8, 76 to 78, 90, 92, 97, 122, 123, 125, 126, 133 to 135, 147, 148, 159, 197, 201, 237, 246 to 249

Abbreviations: ALL, acute lymphoblastic leukemia; AML, Acute myelomonocytic leukemia; CCS, Clinical Classification Software; ICD, International Classification of Diseases; NHL, non-Hodgkin lymphoma.

* Excluded chronic leukemias: ICD-9 204.1, 205.1, 206.1, 207.1.

† CCS, developed by the Healthcare Cost and Utilization Project, groups ICD-9 codes from similar diagnoses into 260 mutually exclusive diagnoses groups (https://www.hcup-us.ahrq.gov/toolssoftware/ccs/CCSUsersGuide.pdf). For the purposes of this research, an admission was considered to involve an infection if it had an ICD-9 code that fit into any of the CCS groups presented in the table.
