# Supplementary material for: How Well Do All Patient Refined–Diagnosis-Related Groups Explain Costs of Pediatric Cancer Chemotherapy Admissions in the United States?
Source: J Oncol Pract. 2016 Apr 26;12(5):e564–75. doi: 10.1200/JOP.2015.010330 (PMC5015448; doi:10.1200/JOP.2015.010330)
Supplement: Publisher's Note [file supp_12_5_e564__index.html]

Publisher's Note 

# How Well Do All Patient Refined–Diagnosis-Related Groups Explain Costs of Pediatric Cancer Chemotherapy Admissions in the United States?

## Publisher's Note

The May 2016 article by Russell et al entitled, "How Well Do All Patient Refined-Diagnosis-Related Groups Explain Costs of Pediatric Cancer Chemotherapy Admissions in the United States?," (J Oncol Pract 12:e564-e575, 2016) was published online April 26, 2016 with an error.

The article now has Gold Open Access with a CC BY-NC-ND 4.0 license. There is now an open lock icon, CC BY graphic, and license information in the sidebar.

This has been corrected as of May 25, 2016.
